# Supplementary material for: Perspective-taking across cultures: shared biases in Taiwanese and British adults
Source: R Soc Open Sci. 2019 Nov 20;6(11):190540. doi: 10.1098/rsos.190540 (PMC6894566; doi:10.1098/rsos.190540)
Supplement: Full list of critical instructions [file rsos190540supp1.docx]

**Supplementary Material 1: Full list of critical instructions**

| English | Mandarin |
| --- | --- |
| Move the large balloon one down from there | 把大氣球向下移一格 |
| Move the large present one up from there | 把大禮物向上移一格 |
| Move the short plant one up from there | 把矮盆栽向上移一格 |
| Move the narrow bottle one up from there | 把窄玻璃瓶向上移一格 |
| Move the narrow cup one up from there | 把窄杯子向上移一格 |
| Move the small ball one up from there | 把小球向上移一格 |
| Move the short jar one down from there | 把矮玻璃罐向下移一格 |
| Move the tall lamp one down from there | 把高檯燈向下移一格 |
| Move the tall stool one up from there | 把高凳子向上移一格 |
| Move the thick cushion one down from there | 把厚座墊向下移一格 |
| Move the thick folder one down from there | 把厚資料夾向下移一格 |
| Move the thin book one up from there | 把薄書向上移一格 |
| Move the thin towel one up from there | 把薄毛巾向上移一格 |
| Move the wide car one down from there | 把寬汽車向下移一格 |
| Move the small tin one down from there | 把小鐵罐向下移一格 |
| Move the wide vase one down from there | 把寬花瓶向下移一格 |
