# Supplementary material for: Perspective-taking across cultures: shared biases in Taiwanese and British adults
Source: R Soc Open Sci. 2019 Nov 20;6(11):190540. doi: 10.1098/rsos.190540 (PMC6894566; doi:10.1098/rsos.190540)
Supplement: Trial sequence for the level-1 visual perspective-taking task [file rsos190540supp2.docx]

**Supplementary Material 2: Trial sequence for the level-1 visual perspective-taking task**

On each trial, participants were cued with a target perspective for 750ms, which informed participants whether they had to make a judgment based on their own or the avatar’s perspective. They then saw a number probe for 750ms, which corresponded to the number of discs in the target perspective. Finally, participants were presented with an image showing an avatar standing in the middle of a room, with discs in front and/or behind the avatar. As soon as this final image was presented, participants were required to verify whether the perspective cue and number probe matched with the image by making a yes/no response (e.g., whether the avatar (he) sees 2 discs). A yes response required a left click on the computer mouse, a no response required a right click on the mouse. The correct response was equally likely to be a yes response versus a no response. A block of 26 practice trials was presented before 4 blocks of 52 test trials were presented. Participants were allowed to take breaks between the test blocks. Participants were instructed to respond as quickly and accurately as possible. If participants did not respond within 2000ms, then the next trial was presented.
