# Supplementary material for: Perspective-taking across cultures: shared biases in Taiwanese and British adults
Source: R Soc Open Sci. 2019 Nov 20;6(11):190540. doi: 10.1098/rsos.190540 (PMC6894566; doi:10.1098/rsos.190540)
Supplement: Fitted models for outputs from the director task [file rsos190540supp3.docx]

**Supplementary Material 3: Fitted models for outputs from the director task**

We attempted to fit a maximal model for all outputs. The maximal model included intercepts from both random effects and slopes for all within-unit factors (i.e., condition | participant, perspective | participant, condition*perspective | participant, condition | grid image, culture | grid image, condition*culture | grid image). Apart from the model for response time data, all other models converged with maximal random effects structures. The fitted model for response time did not contain the condition | grid image and condition*culture | grid image slopes. The fitted models were used to determine the statistical significance of a given main effect or interaction. This was achieved by removing one main effect or interaction term from the fitted model at a time, and comparing the models with versus without a given effect.
