# Supplementary material for: Perspective-taking across cultures: shared biases in Taiwanese and British adults
Source: R Soc Open Sci. 2019 Nov 20;6(11):190540. doi: 10.1098/rsos.190540 (PMC6894566; doi:10.1098/rsos.190540)
Supplement: Fitted models for outputs from the visual perspective-taking task [file rsos190540supp4.docx]

**Supplementary Material 4: Fitted models for outputs from the visual perspective-taking task**

We attempted to fit a maximal model for both response time and error rate. However, neither of the maximal models converged. The fitted model for response time included intercepts for both random effects and slopes for congruency | participant, perspective | participant, congruency *perspective | participant, culture | trial image, perspective | trial image, culture*perspective | trial image. The fitted model for error rate contained intercepts for both random effects and slopes for perspective | participant and perspective | trial image. These fitted models were used to determine the statistical significance of a given main effect or interaction. This was achieved by removing one main effect or interaction term from the fitted model at a time, and comparing the models with versus without a given effect.
